# A Gamma Ornstein–Uhlenbeck model driven by a Hawkes process

**DOI:** 10.1007/s11579-021-00295-0

**Published:** 2021-03-24

**Authors:** Guillaume Bernis, Riccardo Brignone, Simone Scotti, Carlo Sgarra

**Affiliations:** 1 Natixis Assurances, Paris, France; 2 University of Freiburg, Breisgau, Germany; 3 LPSM, Université de Paris (Paris Diderot), Paris, France; 4 Politecnico di Milano, Milan, Italy

**Keywords:** Stochastic volatility, Hawkes processes, Jump clusters, Leverage effect, Exponential affine processes, VIX, Implied volatility for VIX options, C63, G12, G13

## Abstract

We propose an extension of the $\Gamma$-OU Barndorff-Nielsen and Shephard model taking into account jump clustering phenomena. We assume that the intensity process of the Hawkes driver coincides, up to a constant, with the variance process. By applying the theory of continuous-state branching processes with immigration, we prove existence and uniqueness of strong solutions of the SDE governing the asset price dynamics. We propose a measure change of self-exciting Esscher type in order to describe the relation between the risk-neutral and the historical dynamics, showing that the $\Gamma$-OU Hawkes framework is stable under this probability change. By exploiting the affine features of the model we provide an explicit form for the Laplace transform of the asset log-return, for its quadratic variation and for the ergodic distribution of the variance process. We show that the proposed model exhibits a larger flexibility in comparison with the $\Gamma$-OU model, in spite of the same number of parameters required. We calibrate the model on market vanilla option prices via characteristic function inversion techniques, we study the price sensitivities and propose an exact simulation scheme. The main financial achievement is that implied volatility of options written on VIX is upward shaped due to the self-exciting property of Hawkes processes, in contrast with the usual downward slope exhibited by the $\Gamma$-OU Barndorff-Nielsen and Shephard model.

## Introduction

In recent years, the implied volatility indices, such as VIX in the US and V2X in Europe, proved themselves to be key financial instruments for investment strategies, hedging (see [9, 55]) and also indicators of the “stress” on the market [35]. This growing importance has shed light on the peculiar form of the implied volatility dynamics: the occurrence of large variations on very short periods, with a tendency to form clusters of spikes. Moreover, new derivatives products appear, due to market regulation and standardization, like volatility options, with a significant and increasing demand. Implied volatility of these product exhibits an upward slope, this is a clear evidence of market volatility risk aversion pushing people to buy options to cover this risk [53]. The existence of such stylized facts accounts for the emergence of a wide range of financial models taking into account these features. Heston [34] proposes a model where the variance is stochastic and follows a CIR [17] diffusion. By using Poisson processes, Bates [7] adds jumps in asset dynamics, while Sepp [58] includes jumps in both the asset returns and the variance. Duffie et al. [22] generalize the jump-diffusion framework by including a stochastic intensity for the jump processes both in the asset returns and the volatility dynamics, however no self-exciting example is proposed in their paper. Kallsen et al.[43] consider the case where jumps are added in the stock via a time-changed Lévy process. More recently, a large literature develops rough volatility models ([8, 23] and [24]), while Abi Jaber et al. [2] propose a model based on the generalization of affine Volterra processes.

The aim of this paper is to propose a new model for asset pricing able to capture the main stylized features but preserving both mathematical and numerical tractability. Our idea is to build up our model as an extension of the Barndorff-Nielsen and Shephard (BNS) model in order to include jumps clusters in both the volatility and the stock return dynamics. In their celebrated papers Barndorff-Nielsen and Shephard [5, 6] propose a stochastic volatility model of Ornstein–Uhlenbeck type driven by a subordinator; by considering as a concrete specification for the subordinator a compound Poisson process with exponentially distributed jumps size, they obtain a model where both the variance and the log-returns are driven by the same jump process. This type of stochastic volatility model, named $\Gamma$-OU model and originally introduced in order to fit empirical data, was later shown to be suitable for option pricing by Nicolato and Venardos [54]. As pointed out by a large amount of literature (e.g. [59]), single factor models, and, more specifically, the BNS model, are not flexible enough and they are outperformed by multi-factor stochastic volatility models in practice (a multi-factor extension of the BNS model is proposed in [52]). On the other hand, multi-factor models have a main drawback in the huge number of parameters required, giving birth to fitted parameters instability (e.g. [7]).

We propose to extend the $\Gamma$-OU model in order to include jump clustering features. The clustering effect of jumps of the Hawkes [33] process is well suited to take into account the periods of turmoil in the implied volatility, typically observed in implied volatility indices. These clustering features have been studied throughout various financial asset models: see Fiura [28] for FX rates, Hainaut [32] and Jiao et al. [39] for interest rates, Abergel and Jedidi [1], Horst and Xu [36], Zheng et al.[61] for microstructure and limit order books, Errais et al. [25] for credit risk, Jiao et al. [40] for energy prices and Granelli and Veraart [30] for risk premium and contagion. In this paper, we shall insist on the stylized facts related to the clustering effects of the implied volatility indices, such as VIX index, and the related options.

In the model we are going to propose, the jump component of the dynamics is taken into account through a marked Hawkes process, with exponentially distributed jump sizes. Thus, the model has three state variables, the stock, the variance process and the intensity of the jumps. In order to prevent parameters instability, we assume that the intensity process coincides with the variance process up to an additive constant, representing the minimal intensity of jumps arrival. Our model, that will be referred to as $\Gamma$-OU Hawkes volatility model, shares the same number of parameters of $\Gamma$-OU since the constant intensity is replaced by the shifting constant linking the intensity and the variance processes. In spite of this, the $\Gamma$-OU Hawkes model is intrinsically multi-factor, since both variance and intensity are stochastic processes, so it exhibits more flexibility than the $\Gamma$-OU model. Parsimony is preserved by the fact that the two processes coincide up to an additive constant. By exploiting a measure change of Esscher type in a self-exciting setting, as introduced in Jiao et al. [39, 40], we describe the relation between the risk-neutral and the historical dynamics, showing that the $\Gamma$-OU Hawkes framework is stable under this probability change unlike the $\alpha$-stable case in Jiao et al. [39, 41].

We show that it is possible to fit the $\Gamma$-OU Hawkes model to obtain the skew, that is the slope and the left wing of the implied volatility. We then perform a calibration of our model using plain vanilla market data and provide a sensitivity analysis in order to illustrate the flexibility of the model. This sensitivity analysis shows, in particular, that two parameters, namely the speed of mean reversion and the minimal jump intensity, have a small impact on the implied volatility. By using extreme values, we observe that the main impact of both the speed of mean reversion and the minimal jump intensity is detected around the money and consists, roughly speaking, in increasing the smile and preserving the slope for the left side when the minimal jump intensity decreases or the speed of mean reversion increases. This result could appear counterintuitive at first sight since, without jumps, the $\Gamma$-OU Hawkes model, as the BNS, reduces to a Black Scholes diffusion and then the smile is expected to disappear. The explanation is contained in the self-exciting structure of the $\Gamma$-OU Hawkes model since, when the minimal jump intensity decreases, the endogeneity of the jumps increases and that explains why extreme events occur more often.

We also point out that moment explosion does not take place in $\Gamma$-OU Hawkes model. This interesting feature is crucial for several reasons: first, when Monte Carlo simulations are needed in order to perform derivatives evaluation with no close-form solutions available, an infinite variance can constitute a serious drawback. A similar problem arises when considering derivatives with a nonlinear payoff. Third, in dynamic portfolio optimization moment explosion problems can give rise to an infinite value function in many usual frameworks, which represents a main drawback in financial applications.

By focusing on the options written on VIX, the same self-exciting effect gives birth to an upward sloping implied volatility. This upward slope behavior is coherent with market data (e.g. [53]), but is exhibited by very few models in the literature. Nicolato et al. [53] study the implied volatility of variance options for different stochastic volatility models with jumps and show that only inverse-Gamma Ornstein–Uhlenbeck is able to reproduce an upward slope. Indeed, in the case of exponential law for jumps size, the implied volatility for options written on VIX is down-sloping. We then conclude that, in the $\Gamma$-OU Hawkes model, the upward slope is a consequence of self-exciting structure of jumps. We also provide a sensitivity analysis for VIX implied volatility.

We conclude the analysis of options by performing a calibration of the model. The calibration is performed first on Eurostoxx 50 options for two maturities before the COVID crisis. This first set of parameters is exploited for sensitivities analysis. Then, we perform a second round of calibrations on both options on S&P500 and VIX index for a very short maturity (1 month). Calibrations are performed separating S&P500 and VIX options giving birth to two new sets of calibrated parameters. These parameters are similar excepting by the leverage parameter $\rho$ that is close to 0 for VIX option. We link this effect to the definition of VIX index given in the Chicago Board Options Exchange [16] white paper which exploits a static replication valid for continuous paths underlying [21]. Surprisingly the $\Gamma$-OU Hawkes volatility model calibrated on VIX options tries to reproduce the continuous paths forcing a negligible leverage.

Finally, we address the problem of the simulation of the proposed model, which is a very important aspect for its practical applicability. Exact simulation schemes for stochastic volatility models have been first introduced in literature by Broadie and Kaya [12] which show how to simulate exactly the transition density of the Heston (and its extensions with jumps) model. Cai et al. [13] and Li and Wu[49] develop a similar approach for, respectively, SABR [31] and Ornstein–Uhlenbeck stochastic volatility [57] models. The main drawback of those methods is that they rely on the numerical inversion of conditional Laplace transforms which make the simulation schemes slow and hard to implement (see, e.g., [46]). An important advantage of the proposed $\Gamma$-OU Hawkes volatility model is that it can be simulated exactly without resorting to any numerical method. We propose an exact simulation scheme for the model and evaluate the performances by a comparison with the classic Euler scheme.

The rest of the paper is organized as follows. Section 2 details a statistical analysis of VIX values pointing out the existence of jump clusters and giving a first fit to data. In Sect. 3 we introduce the $\Gamma$-OU Hawkes volatility model by providing theoretical results about existence and characterization in a general framework. After presenting the affine properties of the $\Gamma$-OU Hawkes model, we discuss the moment explosion issue. Moreover, we compute the Laplace transform of the quadratic variation of the process involved, and provide a closed form expression for the variance swaps rates. After introducing an Esscher type transform for the present model, we provide a class of equivalent martingale measures. Section 4 is devoted to numerical applications, i.e. European pricing via characteristic function inversion techniques, calibration, perfect simulation and VIX options analysis. Finally, Sect. 5 summarizes the main properties of $\Gamma$-OU Hawkes volatility model, and presents a systematic comparison with both the BNS and the Heston models.

## Clusters in VIX: stylized facts

In this section, we illustrate and discuss the clustering effects of the VIX. A general analysis of this index is performed by Avellaneda and Papanicolaou [4]. This point provides us with an empirical justification of our approach involving clustering effects both on the jumps of the asset price and on the volatility. Moreover, we also see the particular importance of downward jumps. The VIX index represents the square-root of the implied variance extracted from short dated options on the S&P 500 index. Hence, we consider the square of VIX index since it coincides with the variance swap rate, that is a linear functional with respect to time, converging to the quadratic variation of the logarithm of the equity process (see, e.g. [43] for more details).

**Fig. 1:**
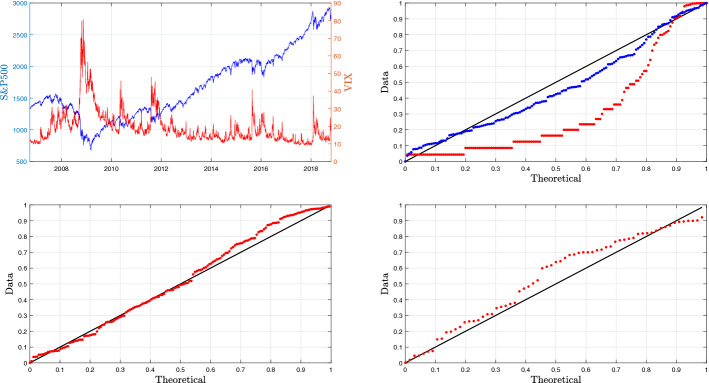
Top-left subplot: joint evolution of S&P and VIX^2^ indices. Top-right subplot: Kolmogorov–Smirnov test for constant (red points) and proportional to the VIX^2^ (blue points) intensity for the jump arrivals. Bottom-left subplot: Kolmogorov–Smirnov test for exponential law for positive fluctuations in VIX^2^. Bottom-right subplot: Kolmogorov–Smirnov test for exponential law for negative fluctuations in S&P

Our sample consists of daily observations from 25-Sep-2006 to 23-Oct-2018, it involves periods of important turmoil (post Lehman Brothers credit crisis after September 2008, European sovereign debt crisis in summer 2011, etc.). The historical time series of the prices of both S&P500 and VIX indices are plotted in the top left panel of Fig. 1. Since the sample starts in a period of mild volatility we can reasonably assume that the Hawkes intensity, at this time, is close to its minimal level, which we denote by ${\underline{\lambda }}$. The jumps are detected as the larger positive fluctuations using the algorithm detailed in Callegaro et al. [14].

The first analysis concerns the distribution of gaps between two jumps in order to confirm that the jumps are clustered. Given the total number of jumps in a given period and assuming a constant intensity, the conditional distribution of gap lengths is uniform. It is now easy to test this hypothesis using a Kolmogorov–Smirnov (K–S) test. The renormalised K–S statistics gives a value of 4.45 that is really large compared to all critical values usually considered: for instance, for a rejection of the null hypothesis at level of 0.01, the critical value is 1.62, for 0.001 it is 1.95. We then reject the Poisson arrival rate of jumps. In contrast, we can test the hypothesis of an intensity proportional to the index VIX^2^ itself and the related renormalised K–S statistics gives a value of 1.04 that is small compared to the critical values usually considered, for instance 1.22 for a significance threshold 0.1. As a consequence, we cannot reject the pure self-excited framework. The top right subplot of Fig. 1 illustrates the result of the goodness of fit. It can be remarked that the values obtained assuming a constant intensity are really far from the ideal diagonal whereas the ones assuming an intensity proportional to the VIX^2^ itself are much closer.

We now turn on the joint analysis of S&P and VIX^2^. We identify, in an independent way, the jump times and the size of the relative increments of S&P and the absolute increments of VIX^2^. Our study shows that almost all large negative jumps of S&P coincide with the positive jumps in VIX^2^, whereas only one half of positive jumps of S&P coincides with the negative ones in VIX^2^. By studying the arrival times of the negative jumps of VIX^2^, we see that they coincide with the very large values of the VIX^2^ itself and follow large positive jumps. We can reproduce this effect with only positive jumps in VIX and an exponential mean-reversion speed. Moreover, the method adopted to identify jumps is unable to detect relatively small jumps (see [14]). In particular, jumps smaller than three standard deviations of the other increments are classified as usual Brownian noise. The fact that we identify almost 2.5 more positive jumps in VIX^2^ than negative jumps in S&P could be explained by the presence of a larger Brownian contribution, covering a part of relatively small jumps. Indeed, we stop our iteration at the fifth loop since the ratio of the variance at the fourth and fifth loop in S&P is 0.975, i.e. really near to 1, showing that the split between the jumps and the Brownian component is done. However, the same ratio for VIX^2^ increments is only 0.83 showing that the Brownian part is certainly overestimated. In order to reach a variance ratio for VIX^2^ similar to the one of S&P more than eleven loops are required. The total number of large fluctuations reaches a frequency of one every ten days that pushes us to cut the Brownian component in the variance process in agreement with the BNS model.

We now focus on the law of jumps sizes in VIX^2^ and S&P. According to the Kou [47, 48] setup we obtain a quite acceptable fit for an exponential law truncated at the threshold used to split increments between jumps and Brownian oscillations. The Kolmogorov–Smirnov test gives values of 1.06 and 1.17 respectively for the positive jumps of VIX^2^ and negative ones of S&P, that could not authorize to reject the exponential law hypothesis. Results on the joint S&P and VIX^2^ analysis are reported in the bottom subplots of Fig. 1.

## The $\Gamma$-OU Hawkes volatility model

As the BNS model, the $\Gamma$-OU Hawkes volatility model is a stochastic volatility model with the same jumps driving both the volatility and the log-return processes, including a leverage effect. We construct the variance process by subtracting a strictly positive constant ${\underline{\lambda }}$ to the Hawkes intensity process (which is bounded from below by ${\underline{\lambda }}$ itself), in this way the variance process exhibits the suited mean-reverting form and can visit all the positive values. Since the model is constructed in order to reproduce the VIX^2^ behavior, which is naturally defined with respect to the risk neutral probability, we start by considering the risk neutral framework, the historical probability will be then introduced in Sect. 3.5.

### Definitions

Let $(\Omega , {{\mathcal {F}}}, {\mathbb {Q}})$ be a probability space supporting a standard Brownian motion $W:=\{W_t\}_{t\ge 0}$ and a marked Hawkes process independent on *W*. The Hawkes process can be characterized by its counting measure $\mu (dt,dz)$ defined on $({\mathbb {R}}^+)^2$ and by the sequence of jump times $\{\tau _i\}_{i\in {\mathbb {N}}}$ and marks $\{Z_i\}_{i\in {\mathbb {N}}}$, representing the jump times and jump sizes respectively. The compensator of $\mu$ can be written as $\lambda _t \theta (dz)dt$, where $\theta (dz)$ is a distribution on the measurable space ${\mathbb {R}}^+$, and the compensated measure is denoted by ${{\tilde{\mu }}} (dz,dt) = \mu (dz,dt) - \lambda _t \theta (dz)dt$. In this paper, we assume that $\theta$ is of exponential type $\theta (dz) := \nu \alpha e^{-\alpha z} dz$. The intensity process reads:

1 $$\begin{aligned} \lambda _t = \lambda _0 + \beta \int _0^t\left( {\underline{\lambda }}-\lambda _s \right) ds + \int _0^t \int _{ {\mathbb {R}}^+} z \, \mu (ds,dz), \end{aligned}$$

where ${\underline{\lambda }}<\lambda _0$ and $\beta$ are positive constants. From now on, we require that the following integrability assumption holds:

#### Assumption 1

The following condition holds:

2 $$\begin{aligned} {\tilde{\beta }}:= \beta - \int _{{\mathbb {R}}^+} z \, \theta (dz) >0. \end{aligned}$$

Under our assumption on the jump size distribution, (2) can be written as ${\tilde{\beta }}:= \beta - \frac{\nu }{\alpha } >0$. This condition could be considered as the usual non-explosion condition for Hawkes processes in a marked Hawkes framework (see [10]).

The stock price process $\{S_t\}_{t\in {\mathbb {R}}^+}$ is defined via its log-return process: $\{X_t\}_{t\in {\mathbb {R}}^+}$: $X_t := \ln (S_t/S_0)$. In order to describe the leverage effect mentioned before, the jump process driving the log-return dynamics is multiplied by $-\rho$, with $\rho > 0$. Without loss of generality, we will assume that the interest and dividend rates are null. We assume that *X* satisfies the following SDE:

$$\begin{aligned} dX_t = \left[ \frac{1}{2} {\underline{\lambda }} - \left( \frac{1}{2} + \int _{{\mathbb {R}}^+} \left( e^{-\rho z} -1 \right) \theta (dz) \right) \lambda _t \right] dt + \sqrt{\lambda _t- {\underline{\lambda }} } \, dW_t - \rho \int _{{\mathbb {R}}^+} z \, \mu (dt,dz), \end{aligned}$$

which under our assumption on the jump size distribution becomes:

3 $$\begin{aligned} dX_t = - \left( \frac{1}{2} \sigma ^2_t - \gamma \lambda _t \right) dt + \sigma _t \, dW_t -\rho \int _{{\mathbb {R}}^+} z \, \mu (dt,dz), \end{aligned}$$

where $\gamma = \frac{\nu \rho }{\rho +\alpha }$ and the variance process is given by $\sigma ^2_t = \lambda _t - {\underline{\lambda }}$

4 $$\begin{aligned} d\sigma ^2_t = -\beta \sigma ^2_t dt + \int _{ {\mathbb {R}}^+} z \mu (dt,dz) . \end{aligned}$$

Note that, thanks to the negative sign in front of the jump term and the domain of $Z_i$, jumps in the log-return process are negative and the compensator is positive. The natural filtration of the point process *N* will be denoted by ${\mathbb {F}}^{N}:=\left\{ {\mathcal {F}}^{N}_t\right\} _{t\ge 0}$, while the natural filtration of $(\lambda , S)$ will be denoted by ${\mathbb {F}}:=\left\{ {\mathcal {F}}_t\right\} _{t\ge 0}$.

### Existence and basic properties

Next, we shall prove existence and regularity of the solution of the SDE, by using the tools of continuous state branching processes with immigration (CBI). In particular, we will show the existence of a strong solution under mild hypotheses including the $\Gamma$-OU Hawkes volatility model. We extend in particular the evolution by assuming a possible infinite activity of the jump component. The $\Gamma$-OU Hawkes volatility model will be obtained under the hypothesis of finite activity and an exponential law for the jump size. The advantage of this generalisation is to provide a unified framework as in Barndorff-Nielsen and Shephard [5, 6], by including both finite and infinite activity. Moreover, the representation exploiting Branching processes features provides valuable insights and ideas for interpretation of the model behaviour. We first write the evolution of the compensated version of the SDE satisfied by the triplet $(\lambda , \sigma ,X)$. We then characterize the evolution as a multi-dimensional exponential affine process where $\lambda$ is an autonomous CBI. A byproduct of this characterization is that our model could be rewritten in the so-called Dawson and Li representation of CBI (see [19, 20, 51]). This last characterization provides access to the ergodic distribution of the process $\lambda$ and $\sigma$. Under hypothesis 1, the process $\lambda$ is exponential ergodic. The law of the invariant distribution and its parameters will be also derived.

#### Lemma 1

(Compensated representation) The triplet $(\lambda , \sigma ,X)$ satisfies the following SDE where the jump component is compensated.

5 $$\begin{aligned} \left\{ \begin{array}{rcl} d\lambda _t &{}=&{} \displaystyle {\tilde{\beta }} \left( \frac{\beta }{ {\tilde{\beta }}}{\underline{\lambda }} -\lambda _t \right) dt + \int _{ {\mathbb {R}}^+} z \, {\tilde{\mu }}(dt,dz)\\ d\sigma ^2_t &{}=&{} \displaystyle {\tilde{\beta }} \left( \frac{\nu }{\alpha {\tilde{\beta }}} {\underline{\lambda }} - \sigma ^2_t \right) dt + \int _{ {\mathbb {R}}^+} z \, {\tilde{\mu }}(dt,dz) \\ dX_t &{}=&{} \displaystyle - \left( \frac{1}{2} \sigma ^2_t - \frac{ \rho }{\alpha } \gamma \lambda _t \right) dt + \sigma _t \, dW_t -\rho \int _{{\mathbb {R}}^+} z \, {\tilde{\mu }}(dt,dz) \end{array} \right. \end{aligned}$$

The proof is a straightforward computation. The crucial point is that the compensator is proportional to $\lambda$. Hereafter, in this section, we assume that the compensated measure satisfies the integrability condition $\int _{{\mathbb {R}}^+} (z \wedge z^2) \theta (dz)< \infty$ (see for instance [50, Section 3.1] and [51, Section 2]). In order to exhibit explicitly this dependency on $\lambda$, we resort to the general theory of continuous state branching processes with immigration and in particular the Dawson and Li representation of the SDE satisfied by $(\lambda , \sigma ,X)$. The next result shows that the couple $(\lambda , X)$ satisfies the Dawson-Li representation of CBI in an extended probability space.

#### Proposition 1

(Dawson–Li representation) There exist an extended probability space where there exist a white noise *W*(*dt*, *du*), defined on $({\mathbb {R}}^+ )^2$, and a compensated Poisson measure ${\tilde{N}}(dt, du, dz)$, defined on $({\mathbb {R}}^+)^3$, with compensator $dt\, du \, \theta (dz)$, such that the triplet $(\lambda , \sigma , X)$ satisfies the SDE

$$\begin{aligned} \lambda _t= & {} \lambda _0 + {\tilde{\beta }} \int _0^t \left( \frac{\beta }{{\tilde{\beta }} }{\underline{\lambda }}-\lambda _s\right) ds + \int _0^{t} \int _0^{\lambda _{s-}} \int _{{\mathbb {R}}^+} z\, {\tilde{N}}(ds, du, dz) \\ \sigma ^2_t= & {} \sigma ^2_0+ {\tilde{\beta }} \int _0^t \left( \frac{\nu }{ \alpha {\tilde{\beta }} } {\underline{\lambda }} - \sigma ^2_s\right) ds + \int _0^{t} \int _0^{\lambda _{s-}} \int _{{\mathbb {R}}^+} z\, {\tilde{N}}(ds, du, dz) \\ X_t= & {} X_0- \int _0^t \left( \frac{1}{2} \sigma _s^2 - \frac{\gamma \rho }{\alpha } \lambda _s \right) ds + \int _0^{t} \int _0^{\sigma ^2_{s}} W(ds, du) \nonumber \\&- \rho \int _0^{t} \int _0^{\lambda _{s-}} \int _{{\mathbb {R}}^+} z \,{\tilde{N}}(ds, du, dz) \, . \end{aligned}$$

This SDE admits a unique solution, which coincides almost surely with the solution of (5).

The main advantage of this representation is to highlight that the speed of mean reversion and level of the intensity between two jumps change due to the self-exciting property. This representation is needed in order to obtain the change of probability result in Sect. 3.5. Moreover, one of the consequences of the previous result is the affine structure of the couple $(\lambda , X)$. The next result characterizes its Fourier–Laplace transform.

#### Proposition 2

(Fourier–Laplace transform) The couple $(\lambda , X)$ is an exponential affine process. That is the Laplace transform of $(\lambda , X)$ satisfies:

$$\begin{aligned} \log \mathrm {E} \Big [ e^{ u X_t +w \lambda _t } \Big ] = u X_0 + \psi _{u,w}(t) \lambda _0 + \phi _{u,w}(t), \end{aligned}$$

for (*u*, *w*) in the domain ${\mathbb {D}}:=\{(u,w) \in {\mathbb {C}} ^2 \, \vert \, \mathfrak {R}{(w)} < \alpha + \rho \mathfrak {R}{(u)} \}$, where $\mathfrak {R}{(\cdot )}$ denotes the real part of a complex number. The functions $\psi$ and $\phi$ satisfy:

$$\begin{aligned} \psi ^{\prime }_{u,w}(t) = R(u, \psi _{u,w}(t)), \quad \phi ^{\prime }_{u,w}(t) = F(u, \psi _{u,w}(t)), \end{aligned}$$

where

6 $$\begin{aligned} R(u,w):= & {} \frac{1}{2} \left( u^2 - u \right) +\gamma u -\beta w +\nu \frac{ w -\rho u}{\alpha +\rho u -w} \nonumber \\ F(u,w):= & {} {\underline{\lambda }} \left[ \beta w - \frac{1}{2} \left( u^2 - u \right) \right] \end{aligned}$$

with starting conditions $\psi _{u,w}(0)=w$ and $\phi _{u,w}(0)=0$. In particular, considering the Fourier transform, the pseudo Riccati operator reads $R(iu,iw)= - \frac{1}{2} \left( u^2 +iu \right) + i \left( \gamma u - \beta w \right) +\nu \frac{ w -\rho u}{\rho u -w - i \alpha }$.

#### Proof

A necessary condition for the Laplace transform to exist is the integrability of the jumps, that is $\int _0^{+\infty }e^{-(u\rho +w-\alpha )z}dz<+\infty$, which requires $\mathfrak {R}{(w)} < \alpha + \rho \mathfrak {R}{(u)}$. In Proposition 3, we shall show that this is actually a sufficient condition. In the following, we consider the operator *R* and *F* only on this domain. The main statement is a direct application of Duffie et al.[22, Proposition 1]. By adopting the notation of Kallsen et al. [43], we can write the differential characteristics (*b*, *c*, *K*) of the process $(\lambda , X)$. Since we consider the compensated version, we do not need to specify any particular truncation function. (5) we obtain

$$\begin{aligned} b = \left( \begin{array}{c} \frac{1}{2}\underline{\lambda } +\left( \gamma - \frac{1}{2}\right) \lambda _{-} \\ \beta \underline{\lambda } - \beta \lambda _{-} \\ \end{array} \right) \quad \quad \quad \quad c = \left( \begin{array}{cc} -\underline{\lambda } + \lambda _{-} &{} 0 \\ 0 &{} 0 \\ \end{array} \right) \\ K(G)= K_1(G) \lambda _{-}, \quad \forall G \in \mathcal {B}^2 ( \mathbb {R}^{+} \times \mathbb {R} ) \quad \text { with } \quad K_1 (dz) = \nu \alpha \exp { (- \alpha z) } \mathrm {1}_{ z>0 } dz \end{aligned}$$

Let’s write $c = c^{0} + c^{1} \lambda _{-}$, $b = b^{0} + b^{1} \lambda _{-}$ and $b = K_0 + K_1 \lambda _{-}$, and recall that the relations between the differential characteristics (*b*, *c*, *K*) and the affine characteristics (*F*, *R*) are the following:

$$\begin{aligned} F(u,w) = \frac{1}{2} (u,w)^{T} c^{0} (u,w) + b^{0} (u,w) + \int \big [ e^{uz} -1 \big ] K_0 (dz), \\ R(u,w) = \frac{1}{2} (u,w)^{T} c^{1} (u,w) + b^{1} (u,w) + \int \big [ e^{wz} -1 \big ] K_1 (dz), \end{aligned}$$

where $(u,w)^{T}$ denotes the transposed of the column vector (*u*, *w*). By remembering that the moment generating function evaluated at *y* of an exponential random variable with parameter $\alpha$ is $y/(y-\alpha )$, we obtain the Riccati system of ODE (6) for $\psi$ and $\phi$. $\square$

#### Remark 1

Proposition 2 can be further extended to obtain the Laplace transform of $\left( X_t, \lambda _t, \int _{0}^{t} X_s ds , \int _{0}^{t} \lambda _s ds\right)$ by using the results in Brignone and Sgarra [11].

For sake of readability, we will write again the generalized Riccati operator in the following form on ${\mathbb {D}}$

7 $$\begin{aligned} R(u,w) = - \frac{ \beta w^2 + p(u) w + u q(u) }{ w - (\alpha + \rho u) } \end{aligned}$$

where *p*(*u*) and *q*(*u*) are the two following quadratic polynomials:

$$\begin{aligned} p(u):= & {} \nu + \frac{1}{2} u \left( 1-u - 2 \gamma \right) - \beta (\alpha +\rho u), \\ q(u):= & {} - \frac{1}{2} \left[ (\alpha +\rho u) \left( 1-u - 2 \gamma \right) + 2 \rho \nu \right] . \end{aligned}$$

The next result is a direct consequence of the affine structure of the model.

#### Corollary 2

(Ergodic distribution) Under Assumption 1, the intensity process $\lambda$ is exponential ergodic and the moment generating function of the invariant distribution is given by

$$\begin{aligned} \mathrm {E}\left[ e^{ w \lambda _{\infty } } \right] = e^{ {\underline{\lambda }} w } \left( 1-\alpha \frac{\beta }{{\tilde{\beta }}} w \right) ^{-\frac{\nu }{\beta }{\underline{\lambda }}} \end{aligned}$$

for $w \in \left( -\infty , \frac{{\tilde{\beta }} }{\alpha \beta } \right)$, that is $\lambda _\infty$ satisfies a Gamma law with scale parameter $\frac{ \alpha \beta }{{\tilde{\beta }} }$, shape parameter $\frac{\nu }{\beta } {\underline{\lambda }}$ and shifting parameter ${\underline{\lambda }}$. The variance process $\sigma ^2$ is also exponential ergodic with a Gamma invariant law with the same parameters and no shift.

#### Proof

According to Jiao et al. [39, Proposition 3.7], we have to compute

$$\begin{aligned} \mathrm {E}\left[ e^{w \lambda _\infty } \right] = \exp \left\{ \int _0^w \frac{F(0,x) }{R(0,x)} dx \right\} = \exp \left\{ \int _0^w \frac{{\underline{\lambda }} \beta x }{ \nu \frac{ x }{\alpha -x} -\beta x } dx \right\} \, \end{aligned}$$

Simplifying, we get:

$$\begin{aligned} \mathrm {E}\left[ e^{w\lambda _\infty }\right]&= \exp \left\{ {\underline{\lambda }} \int _0^w \frac{\beta (\alpha -x)}{\nu -\beta (\alpha -x)} dx \right\} =\exp \left\{ {\underline{\lambda }} w-\frac{{\underline{\lambda }} \nu }{\beta }\int _0^w \frac{\beta }{\nu -\beta (\alpha -x) }dx\right\} \\&=e^{{\underline{\lambda }}w}\left( \frac{\nu -\beta \alpha +\beta w}{\nu -\alpha \beta }\right) ^{-\frac{{\underline{\lambda }} \nu }{\beta }}= e^{{\underline{\lambda }}w}\left( 1-\alpha \frac{\beta }{{\tilde{\beta }}}w\right) ^{-\frac{{\underline{\lambda }} \nu }{\beta }}, \end{aligned}$$

from which the thesis follows. $\square$

### Explicit Laplace transform and moments explosion

Next, we obtain the explicit form of the Laplace transform of the $\Gamma$-OU Hawkes model, but, in order to evaluate its domain, we first deal with the explosion of moments. The next proposition shows that the $\Gamma$-OU Hawkes model has moments of every positive order and the negative moments of orders larger than $u>-\alpha /\rho$.

#### Proposition 3

(Explosion of moments) Under the $\Gamma$-OU Hawkes model, $\mathrm {E}\left[ S_t^u \right] = S_0^u\mathrm {E}\left[ e^{u X_t} \right] < \infty$ if and only if $u>-\alpha /\rho$, with $u\in {\mathbb {R}}$.

#### Proof

In order to perform our analysis on explosion of moments, we need to calculate the function *w*(*u*) such that $R(u, w(u))=0$. According to Keller-Ressel [45, Lemma 3.2], this function is uniquely defined on a maximal domain $I \subset {\mathbb {D}}$ and verifies $w(0)=w(1)=0$. Set out $\Delta (u) := p^2(u) - 4 \beta u q(u)$. We deduce that the set *I* is $\{ u \vert \Delta (u) \ge 0 \}$. Set out $w_\pm (u) := \frac{-p(u) \pm \sqrt{\Delta (u) \;} }{2\beta }$ the two (possibly equal) roots of $\beta x^2 +p(u)x+uq(u)=0$ for $u \in I$. When the two roots are different, the only root which satisfies $w(0) = w(1) = 0$ is $w_{-}(u) = \frac{-p(u) - \sqrt{\Delta (u) \;} }{2\beta }$. Moreover, $w_{-}(u)$ is the only solution which values are always in the domain ${\mathbb {D}}$. In agreement with Keller-Ressel [45, Lemma 3.2], we have for $u \in {\mathbb {R}}$

$$\begin{aligned} f_{+} (u):= & {} \sup \{ w \in {\mathbb {R}}^+ : F(u,w)< \infty \} = \infty \\ r_{+} (u):= & {} \sup \{ w \in {\mathbb {R}}^+ : R(u,w) < \infty \} = \left\{ \begin{array}{ll} \infty &{} \displaystyle \quad u> -\alpha /\rho \\ \alpha +\rho u &{} \displaystyle \quad u\le -\alpha /\rho \end{array} \right. \, , \end{aligned}$$

we then deduce that

$$\begin{aligned} T_\star (u) = \left\{ \begin{array}{lcl} \infty &{} \quad if \quad &{} u>-\alpha /\rho \\ 0 &{} \quad if \quad &{} u \le -\alpha /\rho \end{array} \right. \end{aligned}$$

proving the result. $\square$

We now focus on the explicit solution of the Laplace transform of the couple $(X,\lambda )$. Looking at the ODE satisfied by $\psi$, we remark that it is non-linear but of first order and separable. Then, we can formally solve it as indicated in the following corollary.

#### Corollary 3

(Explicit form for the inverse function of the Laplace transform) We have

8 $$\begin{aligned} t = \int _w^{\psi _{u,w}(t)} \frac{dy}{R(u,y)} = L(\psi _{u,w}(t), u,w)+ H(\psi _{u,w}(t), u, w) \end{aligned}$$

where

$$\begin{aligned} L(\psi _{u,w}(t), u,w):= & {} - \frac{1}{2 \beta } \log \left| \frac{ \beta \, \psi ^2_{u,w}(t) +p(u) \, \psi _{u,w}(t) + u q(u) }{ \beta w^2 +p(u) w + u q(u) } \right| \\ H(\psi _{u,w}(t), u,w):= & {} \left\{ \begin{array}{ll} \frac{p(u) + 2\beta (\alpha + \rho u)}{ 2 \beta \sqrt{\Delta (u) \, } } \log \left| \frac{\big (\psi _{u,w}(t)-w_+(u)\big ) \big (w-w_-(u)\big ) }{\big (w-w_+(u)\big ) \big (\psi _{u,w}(t)-w_-(u)\big )} \right| &{} \; \; \Delta (u) > 0 \\ \frac{p(u) + 2\beta (\alpha + \rho u)}{ \beta } \left[ \frac{1}{ 2\beta w + p(u)} - \frac{1}{ 2 \beta \psi _{u,w}(t) + p(u) } \right] &{} \; \; \Delta (u) = 0 \\ \frac{p(u) + 2\beta (\alpha + \rho u)}{ \beta \sqrt{- \Delta (u) \, }} \left\{ \arctan \left[ \frac{2\beta }{ \sqrt{- \Delta (u) \, }} \left[ \psi _{u,w}(t)+\frac{p(u)}{2\beta } \right] \right] + \right. &{} \; \; \Delta (u) < 0 \\ \quad \quad \quad \quad \quad \quad \quad \quad \left. - \arctan \left[ \frac{2\beta }{ \sqrt{- \Delta (u) \, }} \left[ w+\frac{p(u)}{2\beta } \right] \right] \right\} &{} . \end{array} \right. \end{aligned}$$

#### Proof

The first equality of (8) is a direct consequence of the ODE satisfied by $\psi _{u,w}(t)$, see Proposition 2. We have then to compute explicitly

$$\begin{aligned} t= & {} \int _w^{\psi _{u,w}(t)} \frac{dy}{R(u,y)} = - \int _w^{\psi _{u,w}(t)} \frac{ y - (\alpha + \rho u) }{\beta y^2 + p(u) y + u q(u) } dy \\= & {} - \frac{1}{2\beta } \int _w^{\psi _{u,w}(t)} \frac{ 2\beta y + p(u) }{\beta y^2 + p(u) y + u q(u) } dy + \frac{1}{2\beta } \int _w^{\psi _{u,w}(t)} \frac{p(u) + 2\beta (\alpha + \rho u) }{\beta y^2 + p(u) y + u q(u) } dy\, . \end{aligned}$$

The first integrand has the form $f^\prime (y)/ f(y)$ then we have

$$\begin{aligned}&- \frac{1}{2\beta } \int _w^{\psi _{u,w}(t)} \frac{ 2\beta y + p(u) }{\beta y^2 + p(u) y + u q(u) } dy \\&\quad = - \frac{1}{2\beta } \log \left| \frac{ \beta \left( \psi _{u,w}(t)\right) ^2 + p(u) \psi _{u,w}(t) + u q(u) }{\beta w^2 + p(u) w + u q(u) } \right| \,. \end{aligned}$$

That is the term $L(\psi _{u,w}(t), u,w)$ in (8). For the second term, we need to distinguish three cases, that is if the polynomial $\beta y^2 + p(u) y + u q(u)$ has two real roots, i.e. $\Delta (u) > 0$, only one root, i.e. $\Delta (u)=0$ or complex roots. We first consider the case $\Delta (u) > 0$, i.e. $u\in I$, then $\beta y^2 + p(u) y + u q(u) = \beta (y- w_+(u))(y- w_-(u))$. Then

$$\begin{aligned}&\frac{1}{2\beta } \int _w^{\psi _{u,w}(t)} \frac{p(u) + 2\beta (\alpha + \rho u) }{\beta y^2 + p(u) y + u q(u) } dy \\&\quad = \frac{p(u) + 2\beta (\alpha + \rho u)}{2\beta ^2 (w_+(u) - w_-(u))} \int _w^{\psi _{u,w}(t)} \left[ \frac{1 }{(y- w_+(u)) } -\frac{1 }{(y- w_-(u)) } \right] dy\,. \end{aligned}$$

By splitting and integrating the integral and recalling that $w_+(u) - w_-(u) = \sqrt{\Delta (u) \;} / \beta$, we obtain

$$\begin{aligned}&\frac{1}{2\beta }\int _w^{\psi _{u,w}(t)} \frac{p(u) + 2\beta (\alpha + \rho u) }{\beta y^2 + p(u) y + u q(u) } dy \\&\quad = \frac{p(u) + 2\beta (\alpha + \rho u)}{2\beta \sqrt{\Delta (u) \;} } \log \left| \frac{ (\psi _{u,w}(t)- w_+(u)) \, (w- w_-(u)) }{(w- w_+(u)) \, (\psi _{u,w}(t)- w_-(u))} \right| \, . \end{aligned}$$

Suppose now that the two solutions coincide that is $\Delta (u)=0$ and $w_-(u) = w_+(u) = - p(u)/(2\beta )$. Then, we have

$$\begin{aligned}&\frac{1}{2\beta }\int _w^{\psi _{u,w}(t)} \frac{p(u) + 2\beta (\alpha + \rho u) }{\beta y^2 + p(u) y + u q(u) } dy\\&\quad = \frac{p(u) + 2\beta (\alpha + \rho u)}{2\beta ^2 } \left[ \frac{1}{w + \frac{p(u)}{2\beta }} - \frac{1}{\psi _{u,w}(t) + \frac{p(u)}{2\beta }} \right] \, . \end{aligned}$$

Finally, when $\Delta (u)<0$, we have

$$\begin{aligned} \frac{1}{2\beta }\int _w^{\psi _{u,w}(t)} \frac{p(u) + 2\beta (\alpha + \rho u) }{\beta y^2 + p(u) y + u q(u) } dy&= \frac{p(u) + 2\beta (\alpha + \rho u) }{ \beta \sqrt{-\Delta (u)} } \Bigg [ \arctan \left( \frac{2\beta \psi _{u,w}(t) + p(u)}{\sqrt{-\Delta (u)}} \right) + \\&- \arctan \left( \frac{2\beta w + p(u)}{\sqrt{-\Delta (u)}} \right) \Bigg ]\,. \end{aligned}$$

From which the thesis follows. $\square$

#### Remark 2

The function *H* coincides, up to some constants, to the corresponding function of the Heston model. The function *L*, that does not appear in Heston case, is the logarithm of a quadratic function of $\psi$.

### Variance swap and VIX

Next, we aim to provide closed form expressions for the variance swap rates. The VIX index could then be obtained easily. VIX is the forecasted average volatility of S&P during the next month, the equivalent index based on Eurostoxx 50 is called V2X. In the rest of the paper, the term “VIX” is used broadly to refer to VIX or V2X or all other volatility indices based on a particular underlying, where no risk of confusion exists. The next proposition, gives the explicit form of the variance swap rates.

#### Proposition 4

(Variance swap rates) Under $\Gamma$-OU Hawkes volatility model, the variance swap rate at time *t* with a time to maturity $t+T$ reads

9 $$\begin{aligned} K_{t}(T) := \mathrm {E} \Big [ [X]_{T+t} - [X]_{t} \big \vert {\mathcal {F}}_t \Big ] = \Phi _1 (T) \lambda _t + \Phi _0(T) \end{aligned}$$

where

10 $$\begin{aligned} \Phi _1 (T)= & {} \left[ 1 + 2 \nu \left( \frac{\rho }{\alpha }\right) ^2 \right] \frac{ 1- e^{-{\tilde{\beta }} T} }{{\tilde{\beta }}} \nonumber \\ \Phi _0 (T)= & {} \frac{ {\underline{\lambda }} \nu }{{\tilde{\beta }} } \left[ \frac{1}{\alpha }+ 2 \beta \left( \frac{\rho }{\alpha } \right) ^2 \right] \, T - \beta {\underline{\lambda }} \left[ 1+ 2 \nu \left( \frac{\rho }{\alpha } \right) ^2 \right] \frac{ 1- e^{-{\tilde{\beta }} T } }{{\tilde{\beta }}^2 } \end{aligned}$$

#### Proof

Recalling that the $\Gamma$-OU Hawkes volatility model belongs to exponential affine class, we can obtain the Laplace transform of the quadratic variation thanks to Kallsen et al. [43, Lemma 4.2]. It yields that, for $\mathfrak {R}{(u)} \in {\mathbb {R}}^-$,

$$\begin{aligned} \log \mathrm {E}\left[ \left. e^{ u [X]_{T+t} } \right| {\mathcal {F}}_t \right] = \Psi _0 (T,u) + \Psi _1 (T,u) \lambda _t + u [X]_t \end{aligned}$$

where $\Psi _{0} (T,u) =\int _0^T \beta {\underline{\lambda }} \Psi _{1}(s,u) ds - {\underline{\lambda }} \, u \, T$ and $\Psi _1$ satisfies

$$\begin{aligned} \frac{\partial \Psi _1}{\partial T}(T,u)= & {} -\beta \Psi _1(T,u) + u + \int _0^\infty \left( e^{\Psi _1(T,u) z +u \rho ^2 z^2} -1 \right) \nu \alpha e^{-\alpha z} dz \\= & {} -\beta \Psi _1(T,u) + u + \frac{\nu \alpha }{2 \sqrt{-\rho ^2 u \, }} \; {\mathcal {U}}\left( \frac{1}{2},\frac{1}{2},\frac{\left[ \alpha -\Psi _1(T,u)\right] ^2}{-4\rho ^2 u}\right) - \nu \end{aligned}$$

with $\Psi _1(0,u)=0$ where ${\mathcal {U}}$ denotes the confluent hypergeometric function of the second kind. We can obtain the expected value of the quadratic variation by differentiation of the Laplace transform and setting $u=0$. As in Kallsen et al. [43, Lemma 4.2], we obtain, under Assumption 1,

$$\begin{aligned} \mathrm {E}\Big [ [X]_{T+t} \big \vert {\mathcal {F}}_t \Big ] = \Phi _0 (T) + \Phi _{1}(T) \lambda _t + [X]_t \end{aligned}$$

where $\Phi _i(T) := - \frac{ \partial \Psi _{i}}{\partial u} (T,0)$. In order to obtain the explicit form of $\Phi _1(T)$, we differentiate (10) and, taking $u=0$, we obtain the differential equation satisfied by $\Phi _1$. It reads

$$\begin{aligned} \Phi _1^{\prime }(T)= & {} -\beta \Phi _1(T) + 1 + \nu \frac{ \Phi _1(T) }{\alpha } + 2 \nu \left( \frac{\rho }{\alpha } \right) ^2 = - {\tilde{\beta }} \Phi _1(T) + 1+ 2 \nu \left( \frac{\rho }{\alpha } \right) ^2 \, . \end{aligned}$$

By solving the previous linear equation and recalling the initial condition $\Phi _1(0)=0$, we obtain the explicit form for $\Phi _1(T)$ given in the statement. Similarly, differentiating the relation satisfied by $\Psi _0$ and taking $u=0$, we obtain $\Phi _0(T) =\int _0^T \beta {\underline{\lambda }} \Phi _{1}(s) ds + {\underline{\lambda }} \, T$ and a direct integration gives the explicit form for $\Phi _1(T)$ in the statement. $\square$

We can obtain the following corollary since the VIX index could be expressed as the square root of the variance swap rate with maturity one month (see e.g. [4, 41, 53, 55] and references therein).

#### Corollary 4

Let *T* equal to one month, i.e. $T:= 1/12$, then VIX index reads $VIX_t =\sqrt{ \Phi _1 (T) \lambda _t + \Phi _0(T)}$. Moreover, the forward rate of VIX index reads

11 $$\begin{aligned}&{\mathbb {E}}\left[ \sqrt{\Phi _1 (T) \lambda _t + \Phi _0(T)} \right] \nonumber \\&\quad = \frac{1}{2 \sqrt{\pi }} \int _0^\infty \frac{1}{x^{3/2}} \Big (1- \exp \big \{ \lambda _0 \, \psi _{0, -x \, \Phi _1 (T)}(t) + \phi _{0, -x \, \Phi _1 (T)}(t) -x \Phi _0(T) \big \} \Big ) dx\, . \end{aligned}$$

The proof of the last statement is based on the Laplace transform of square root function using the Laplace transform given in Proposition 2. Equation (11) is almost closed given the result of Corollary 3.

### Change of probability

In this section, we investigate the change of probability in the framework defined above. One of the main advantages of the integral representation detailed in Proposition 1 is that it gives rise to a natural extension of the Esscher transform in the jump clustering framework. The main result is detailed in the following proposition showing that the class of $\Gamma$-OU Hawkes models is stable under a self-exciting Esscher type change of probability (see [39, 40] for similar results in the $\alpha$-stable case, which is not stable under the same kind of probability change). We point out that the Esscher transform that we are going to introduce is not the Esscher transform for the semimartingale $S_t$, as defined by Kallsen and Shiryaev [44], but it is the Esscher transform of the driving Hawkes process, strictly analogous to the transform defined by Nicolato and Venardos [54, eq. 3.14]. For a critical presentation of the Esscher transform for BNS models we refer to Hubalek and Sgarra [37]. In this section, in order to avoid ambiguity, we add a superscript ${\mathbb {P}}$ or ${\mathbb {Q}}$ on the various quantities of (5) depending on the reference probability.

#### Proposition 5

(Self-exciting Esscher transform) Let $(\lambda ,\sigma , X )$ be as in Proposition 1 under the risk neutral probability ${\mathbb {Q}}$. Fix $(\eta , \xi ) \in {\mathbb {R}} \times (-\alpha ^{{\mathbb {Q}}},\, \infty )$ and define

$$\begin{aligned} U_t:= \eta \int _0^t \int _0^{\sigma ^2_s} W(ds,du)+ \int _0^t \int _0^{\lambda _{s-}} \int _{{\mathbb {R}}^+} \left( e^{- \xi z } -1 \right) \, {\tilde{N}}(ds,du,dz) \end{aligned}$$

Then, the Doléans–Dade exponential ${\mathcal {E}}(U)$ is a martingale and the probability measure ${\mathbb {P}}$ defined by $\left. d{\mathbb {P}} / d{\mathbb {Q}} \right| _{{\mathcal {F}}_t} :={\mathcal {E}}(U)_t$ is equivalent to ${\mathbb {Q}}$. Moreover, under ${\mathbb {P}}$, the couple $(\lambda ,X)$ satisfies the evolution of exponential affine class (see Eq. 5) with parameters

$$\begin{aligned} \alpha ^{ {\mathbb {P}}}:= & {} \alpha ^{ {\mathbb {Q}}} +\xi , \quad \quad \nu ^{ {\mathbb {P}}} := \nu ^{ {\mathbb {Q}}}\frac{\alpha ^{ {\mathbb {Q}}} + \xi }{ \alpha ^{ {\mathbb {P}}} } \quad \quad {\tilde{\beta }}^{ {\mathbb {P}}} := {\tilde{\beta }}^{ {\mathbb {Q}}} - \frac{\nu ^{ {\mathbb {Q}}} \alpha ^{ {\mathbb {Q}}} }{( \alpha ^{ {\mathbb {Q}}} + \xi )^2 } \left[ 1 - \frac{\nu ^{ {\mathbb {Q}}} \alpha ^{ {\mathbb {Q}}} }{ ( \alpha ^{ {\mathbb {Q}}} )^2 } \right] , \end{aligned}$$

and the dynamics with respect to ${\mathbb {Q}}$ takes the following form:

$$\begin{aligned} dX_t= & {} - \left[ \left( \frac{1}{2} + \eta \right) \sigma ^2_t - \rho \lambda _{t-} \nu ^{ {\mathbb {Q}}} \left( \frac{1}{ \rho + \alpha ^{{\mathbb {Q}}} } - \frac{1}{ (\alpha ^{ {\mathbb {Q}}} + \xi )^2 } \right) \right] dt \\&+ \sigma _t dW^{ {\mathbb {P}}}_t - \rho \int _{{\mathbb {R}}^+} z \, {\tilde{\mu }}^{ {\mathbb {P}}} (dt,dz),\\ d\lambda _t= & {} {\tilde{\beta }}^{{\mathbb {P}}} \left( \frac{\beta }{ {\tilde{\beta }}^{{\mathbb {P}}} }{\underline{\lambda }} -\lambda _t \right) dt + \int _{ {\mathbb {R}}^+} z \, {\tilde{\mu }}^{ {\mathbb {P}}}(dt,dz). \end{aligned}$$

#### Proof

First, we remark that $\int _{{\mathbb {R}}^+} e^{- \xi z } \; \theta ^{{\mathbb {P}}}(dz)<\infty$, since $\mathfrak {R}{(\xi )} \in (-\alpha ^{{\mathbb {Q}}},\, \infty )$. It is easy to show that the triplet $(\lambda , X, Y)$, where $Y:= {\mathcal {E}}(U)$, is Markovian and exponential affine by applying the same argument of Proposition 1. Thanks to the integrability property we can then apply Kallsen and Muhle-Karbe [42, Corollary 3.9] and this will imply that *Y* is a martingale, that ${\mathbb {P}}$ exists and it is equivalent to ${\mathbb {Q}}$. We have easily that $dY_t = Y_{t-} dU_t$. For the second statement, let $f\in C^2_b({\mathbb {R}}^+\times {\mathbb {R}})$, we apply Itō formula to $H_t:=f(\lambda _t , X_t)\, Y_t$. Let’s denote by $f_\lambda$ (resp. $f_X$) the first derivative of *f* with respect to $\lambda$ (resp. *X*). A standard but tedious computation gives

$$\begin{aligned} dH_t= & {} \text { Local Martingale } + Y_{t-} \Bigg \{ \frac{1}{2} f_{XX}(\lambda _t ,X_t)\, \lambda (t) \\&+f_\lambda (\lambda _{t-}, X_{t-}) \, \left[ \left( \beta {\underline{\lambda }} - {{\tilde{\beta }}}^{{\mathbb {Q}}} \lambda _{t-} \right) + \lambda _{t-} \int _{{\mathbb {R}}^+} z \left( e^{- \xi z } - 1 \right) \, \theta ^{{\mathbb {Q}}} (dz) \right] \\&+ f_X(\lambda _{t-}, X_{t-}) \left[ - \left( \frac{1}{2} + \eta \right) \sigma ^2 _t + \rho \lambda _{t-} \int _{{\mathbb {R}}^+} z \left( e^{- \xi z } -1 \right) \theta ^{{\mathbb {Q}}} (dz) \right] \\&+ \lambda _{t-} \int _{{\mathbb {R}}^+} \Big [ f(\lambda _{t-} + z, X_{t-}- \rho z ) - f(\lambda _{t-}, X_{t-} ) - z f_\lambda (\lambda _{t-}, X_{t-}) \\&\quad \quad \quad \quad \quad \quad \quad \quad + \rho z f_X(\lambda _{t-}, X_{t-}) \Big ] \, e^{- \xi z } \, \theta ^{{\mathbb {Q}}} (dz) \Bigg \} \, dt \end{aligned}$$

By identifying the terms, we obtain the evolution of $(\lambda ,X)$ under ${\mathbb {P}}$. $\square$

## Numerical applications

This section deals with numerical studies of the $\Gamma$-OU Hawkes volatility model. In the first part, we show how to price European call options using characteristic function inversion techniques. A calibration on a recent set of market data is proposed. Moreover, we estimate the behaviour of the slope of the implied volatility as time to maturity goes to zero and show that it exhibits a power decay with parameter 0.2. The second part deals with options written on VIX index. The main result is that VIX implied volatility increases with strike.Nicolato et al. [53] have shown that $\Gamma$-OU model exhibits a VIX implied volatility decreasing with the strike. We deduce that the increasing VIX implied volatility is a consequence of the self-exciting structure. Then, we present some results on the calibration on S&P 500 and VIX options on the same date and maturity and compare the resulting parameters. In the third part we show how to simulate efficiently the $\Gamma$-OU Hawkes model. In particular, we will show that the model can be simulated exactly, allowing for unbiased estimation of derivatives prices. Finally, we study numerical efficiency of the proposed method through a comparison with the Euler simulation scheme.

Computations are done using Matlab^®^(Version R2019b) in Microsoft Windows 10^®^running on a machine equipped with Intel(R) Core(TM) i7-9750HQ CPU @2.60GHz and 16 GB of RAM.

### European option pricing and calibration

In Sect. 3.3 we obtained the Laplace transform of $(X, \lambda )$ in terms of the solution of an ODEs system. The characteristic function of log-returns under the proposed model is given by $\Upsilon (u):=\mathrm {E}\left[ e^{i u X_T}\right]$ and can be obtained by Proposition 2. This result opens the doors to option pricing via standard characteristic function inversion algorithms such as, for example, FFT [15] and COS [26] methods. The latter has the advantage to present exponential convergence to the true solution, while preserving linear computational complexity. The main consequence is that option prices can be estimated through a smaller number of evaluations of the characteristic function, which is particularly important when it has to be computed through time consuming numerical techniques such as solution of ODEs systems (as in the present case). See Brignone and Sgarra [11] for more details. Hence, given an initial price $S_0$, a strike *K* and maturity *T*, European option prices are computed as

12 $$\begin{aligned} \mathrm {E}[(S_T - K)^+] = \int _{-\infty }^{\infty } (S_0e^{x} - K)^+f(x)dx \approx \int _{a}^{b} (S_0e^{x} - K)^+f(x)dx \end{aligned}$$

where

13 $$\begin{aligned} f(x) = \sum _{k=1}^{\infty } F_k \cos \left( k \pi \frac{x - a}{b - a}\right) + \frac{1}{b - a} \approx \sum _{k=1}^{N-1} F_k \cos \left( k \pi \frac{x - a}{b - a}\right) + \frac{1}{b - a}, \end{aligned}$$

$F_k = \frac{2}{b - a} \mathfrak {R}\left( \Upsilon \left( \frac{k \pi }{b -a}\right) \; \exp \left( -i \frac{k a \pi }{b -a }\right) \right)$ and $[a, b] \in {\mathbb {R}}$ is chosen such that: $\int _{a}^{b} e^{i u x}f(x) ds \approx \int _{-\infty }^{\infty } e^{iux}f(x) dx.$ Following Fang and Oosterlee [26] we set

14 $$\begin{aligned} a = c_1 - L\sqrt{c_2 + \sqrt{c_4}}, \quad b = c_1 + L\sqrt{c_2 + \sqrt{c_4}} \end{aligned}$$

where *L* can be chosen arbitrary large and $c_i$ denotes the *i*-th cumulant of $\ln \left( \frac{S_T}{K}\right)$. Since the model is affine, cumulants can be computed analytically, for this purpose, we adapt the procedure outlined in Feunou and Okou [27]. According to Fang and Oosterlee [26], if $L= 10$, (14) gives a truncation error around $10^{-12}$, which is negligible for our purposes. Given European options prices, the corresponding implied volatility can be computed by inverting the Black–Scholes formula.

**Fig. 2:**
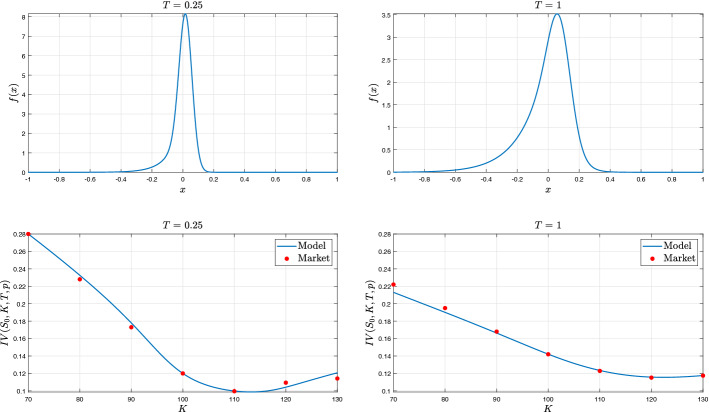
Probability density functions of $X_T$ and implied volatilities for short and long maturities for the calibrated parameters in Table 1 (Set A)

Equipped with an efficient procedure for computing the implied volatility surface, we calibrate our model on the Eurostoxx 50 market data of the 19 November 2019. We minimize the differences (in absolute value) between market and model implied volatilities. The resulting parameters are displayed in Table 1 (Set A). Calibrated risk neutral density and implied volatility are plotted for different maturities in Fig. 2. We remark that the density functions have a left tail heavier than the right one especially for the shorter maturity.

**Fig. 3:**
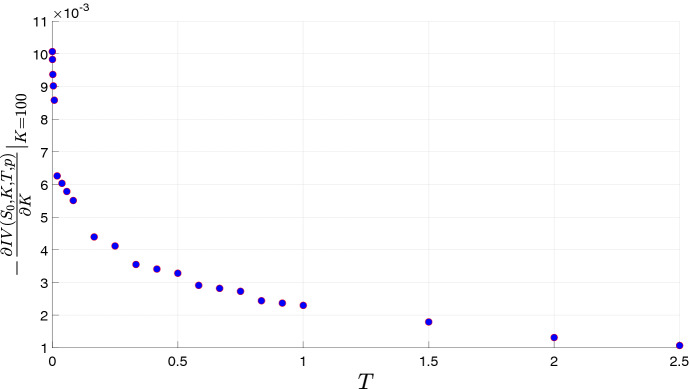
Behavior of the slope of implied volatility skew as function of the maturity over a range from 5 min to 2.5 years. Model parameters as in Table 1 (Set A)

We now focus on the slope of ATM implied volatility, i.e. the derivative of implied volatility with respect to the at the money strike, and its dependency on the maturity. Figure 3 confirms that the implied volatility exhibits a steeply negative ATM slope. This phenomenon is magnified for short maturities over an extremely large range, down to 5 minutes that could be considered as a limit of microstructure. According to empirical studies, we test if the slope of implied volatility skew exhibits a power decay with time to maturity. The ATM implied volatility slope in the $\Gamma$-OU Hawkes volatility model has a power decay behaviour with parameter $-0.20$ and an $R^2$ coefficient of determination of 0.81. We stress that in the Heston model it presents a different behavior: it converges to a finite value as time to maturity goes to zero. Then, the behaviour of the $\Gamma$-OU Hawkes volatility model is extremely different from that of the Heston model (for short maturities) and replicates better the empirical facts.

**Fig. 4:**
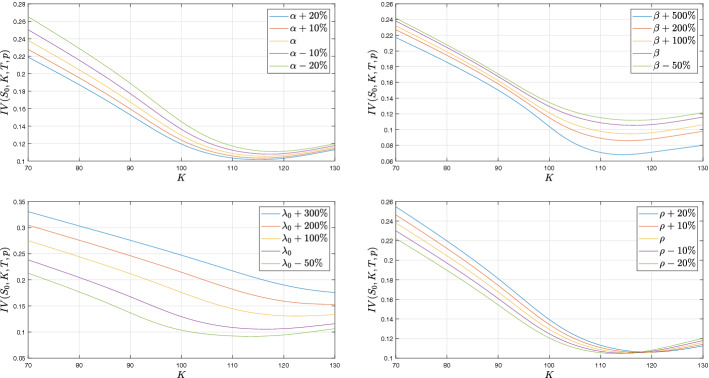
Implied volatilites for varying model parameters. Option contract parameters: $S_0=100$, $T= 0.5$. Model parameters are as in Table 1 (Set A), initial volatility $\sigma _0^2$ is fixed at 0.0079

Next, we focus on the sensitivity of the implied volatility with respect to model parameters. In Fig. 4 we show the model implied volatility for different sets of parameters. We decide to consider $\sigma _0$ as an initial condition for the process. Then sensitivities with respect to ${\underline{\lambda }}$ are obtained shifting both ${\underline{\lambda }}$ and $\lambda _0$ by the same value. We will focus on the impact of a change of a parameters on the level and the slope of implied volatility for negative moneyness, the position of the minimum of the implied volatility and the smile (i.e. convexity around this minimum). We first remark that an important change of ${\underline{\lambda }}$ or $\beta$ is needed to distinguish their sensitivities. We will see that these parameters impact more the implied volatility for options written on VIX. For extreme changes on ${\underline{\lambda }}$, we observe that the main impact of an increase in ${\underline{\lambda }}$ is that the position of the minimum of implied volatility moves on right and the implied volatility goes up, the slope for negative moneyness is unchanged. For extreme changes on $\beta$, we observe that the main impact of an increase in $\beta$ is to accentuate the smile for positive moneyness. There is also a small negative effect on the level of implied volatility. The position of the minimum and the slope are roughly unchanged. A small change on $\alpha$ and/or $\rho$ has an important effect on the slope with opposite directions, i.e. a positive change in $\alpha$ (resp. $\rho$) decreases (resp. increases) the steepness of the slope. Convexity is roughly unchanged in both the cases. $\rho$ has a positive impact on the position of the minimum whereas $\alpha$ has no effect.

### Options on VIX

**Fig. 5:**
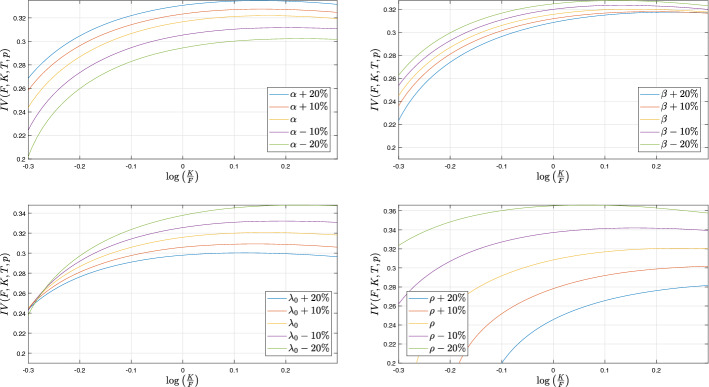
Implied volatility of the options on volatility for varying model parameters. Option contract parameters: ${\hat{T}} = \frac{1}{12}$, $T= 1$. Model parameters are as in Table 1 (Set A), ${\underline{\lambda }}$ is computed such that $\sigma ^2_0$ is fixed at 0.0079

We focus now on options written on VIX index that coincides with the square root of the variance swap rate. Of course, this analysis also applies to the equivalent index based on the Eurostoxx 50, the V2X index. According to Proposition 4, the variance swap rate is affine in $\lambda$ and given by (9). Call and Put options on VIX or V2X are very popular with trading volumes increasing year after year. The prices of these options is generally expressed in terms of Black–Scholes implied volatility even if the structure of the model is far from log-normality and the index itself is not computed in a linear way. These options will be referred to as *options on volatility*, whatever the underlying index (VIX or V2X). In order to use Black–Scholes formula, we need to define the options and their associated forward contract, thanks to (9), we have

$$\begin{aligned} F_{VIX}( T)&= \mathrm {E}\left[ \sqrt{\Phi _1({\hat{T}}) \lambda _{T} + \Phi _0({\hat{T}})}\right] ,\quad \\ C_{VIX}(T ,K)&= \mathrm {E}\left[ \left( \sqrt{\Phi _1({\hat{T}}) \lambda _{T} + \Phi _0({\hat{T}})} - K\right) ^+\right] \end{aligned}$$

where the variance time to maturity ${\hat{T}} := \frac{1}{12}$ is one month in coherence with the definition of VIX and V2X , the expiry of the option is denoted by *T*. Thus, the underlying volatility swap goes from *T* to $T+{\hat{T}}$. Figure 5 shows the implied volatility of options written on V2X. The yellow curve is obtained using the calibrated parameters in Table 1 (Set A). We observe that the implied volatility is increasing and then coherent with the usual behavior of VIX implied volatility. Comparing with the exponential law case studied in Nicolato et al.[53], we highlight that the self-exciting property of the $\Gamma$-OU Hawkes volatility model changes the slope of VIX implied volatility from negative to positive. Nicolato et al. [53] obtain a positive slope but they need to consider an inverse-gamma law with $\nu =1.2$ and then a model with infinite variance. The $\alpha$-Heston model by Jiao et al. [41], which accounts both for power decay and self-exciting features, exhibits an implied VIX volatility which is convex around the money but without any moment larger than the first one. From a financial point of view, the increasing shape of the VIX smile can be explained by the important role played by the options on VIX for high strikes, i.e. protection against turmoils in the equity markets. The strong demand for this protection is responsible for the high levels of the implied volatility for elevated strikes. Figure 5 also details the sensitivities of the implied volatility of options written on VIX with respect to $\alpha$, $\beta$, ${\underline{\lambda }}$ and $\rho$. First, we note that a change in $\alpha$ impacts the level of implied volatility, acting as a translation of the smile. Without surprise the influence of $\rho$ is the more glaring. Second, we can observe that the VIX implied volatility also exhibits a sensitivity with respect to $\beta$ and ${\underline{\lambda }}$ for standard perturbations. Given the small sensitivity of implied volatility of the underlying with respect to $\beta$ and ${\underline{\lambda }}$, it is natural to choose $\beta$ and ${\underline{\lambda }}$ to fit the smile of the VIX volatility.

### Calibration on S&P500 and VIX options

Next, we calibrate the proposed model on S&P500 and VIX options. We find model parameters minimizing the difference in absolute value between the market and model implied volatilities (1 month maturity). Resulting parameters are reported in Table 1, with parameter set B resulting from the calibration on S&P500 options and set C resulting from the calibration on options on VIX. Results show that the $\Gamma$-OU Hawkes volatility model calibrates well on S&P500 and VIX options separately. We highlight that the options sets used for the calibration refer to a crisis period, i.e. November 6 2020, for both the COVID-19 pandemic and the close presidential contest in US. However, a joint calibration does not work well. It is easy to remark that the main issue is related to the leverage parameter $\rho$. The other parameters are similar, in particular comparing with the values estimated out of the crisis (i.e. set A).

In particular we remark that the calibrated leverage $\rho$ is negligible for VIX options. This effect is probably due to the VIX definition. As indicated in the Chicago Board Options Exchange [16] white paper, VIX index is computed as the square root of the static replication of Variance Swap rates. This static replication is introduced in Demeterfi et al. [21] and is based on the crucial hypothesis of continuous paths of the underlying. In Demeterfi et al. [21, pp. 29-35] the impact of jumps on the underlying is detailed showing that a bias exists as long as underlying jumps. In our model, jumps in the underlying disappear as long as the leverage parameter $\rho$ goes to zero. It is then quite natural that a joint S&P500 and VIX calibration is unattainable in practice. Moreover, the VIX is based on an average of short dated options of different expiries and not a single expiry. More surprisingly, the two distinct calibrations on the same day give similar parameters with the substantial exception of the leverage $\rho$. We could conclude that the $\Gamma$-OU Hawkes volatility model fits the behaviour of implied volatilities on both S&P500 and VIX albeit a more detailed theoretical analysis of the jumps corrections is needed to confirm this goodness.

**Fig. 6:**
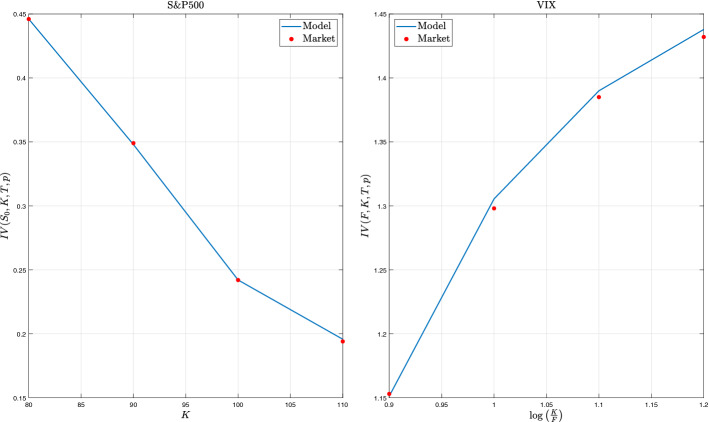
Separate calibration of option on S&P500 (left subplot) and options on VIX (right subplot)

**Table 1:** Calibrated parameters. Parameter set A is calibrated on options Eurostoxx (9 November 2019), Parameter set B is calibrated on options on S&P500 (6 November 2020), Parameter set C is calibrated on options on VIX (6 November 2020). For sake of completeness, we indicate the initial value of the intensity $\lambda _0 = \sigma _0^2 + {\underline{\lambda }}$

Set	${\underline{\lambda }}$	$\alpha$	$\nu$	$\beta$	$\rho$	$\sigma _0^2$	$\lambda _0$
A	1.6372	50.1123	0.9040	1.1819	2.8853	0.0079	1.6451
B	2.9851	16.2035	1.6805	4.2392	1.1106	0.0290	3.0141
C	2.9731	14.4447	2.0494	3.4374	0.0498	0.0186	2.9917

### Exact simulation and its performance

We start by pointing out that the model simulation crucially depends on the Hawkes process. Several exact simulation schemes have been proposed in literature for the simulation of this kind of process. Gonzato et al. [29] provides an extensive literature review and finds the exact simulation scheme proposed by Dassios and Zhao [18] is the most efficient, being the fastest among the exact methods. Hence, we decide to simulate the Hawkes process using the exact method of Dassios and Zhao [18]: given an initial date $t_0 = 0$, a final date *T*, an initial value for $\lambda _0$ and the parameters of the model, the algorithm allows to obtain a sample of the quadruplet $\left( N_T, \{\tau _k\}_{k=1}^{N_T}, \{\lambda _{\tau _k}\}_{k=1}^{N_T}, \{Z_{\tau _k}\}_{k=1}^{N_T}\right)$, where $N_T$ is the total number of jumps in the period [0, *T*], $\tau _k$ is the $k-$th jump time, $Z_{\tau _k}$ is the $k-$th jump size which has an exponential distribution with parameter $\alpha$. Given the quadruplet, we can compute:

$$\begin{aligned} \lambda _T= {\underline{\lambda }}+ (\lambda _{\tau _{N_T}}-{\underline{\lambda }})e^{-\beta (T - \tau _{N_T})}, \quad \int _{0}^{T} \lambda _s ds = -\frac{\lambda _T- \lambda _0 - \beta {\underline{\lambda }}T - \sum _{k=1}^{N_T} Z_{\tau _k}}{\beta }, \end{aligned}$$

moreover, $$\int _{0}^{T} \sigma _s^2 ds = \int _{0}^{T} \lambda _s ds - {\underline{\lambda }} T$$ and

15 $$\begin{aligned} X_T \Big | \lambda _T, \int _{0}^{T} \lambda _s ds \sim {\mathcal {N}}(m, s^2) \end{aligned}$$

where

16 $$\begin{aligned} m&= X_0 + \gamma \int _{0}^{T} \lambda _s ds - \rho \sum _{k=1}^{N_T} Z_{\tau _k} - \frac{1}{2}\int _{0}^{T} \sigma _s^2 ds, \quad s^2 = \int _{0}^{T} \sigma _s^2 ds. \end{aligned}$$

Hence, is possible to simulate $X_T$ given $\lambda _0$ exactly and efficiently (no numerical methods or approximations are required at any step), implying that unbiased estimators for path independent derivatives can be obtained. Moreover, is also possible to extend the methodology in order to generate paths for log-returns process observed at a discrete time grid, we summarize the procedure in Algorithm 1. 
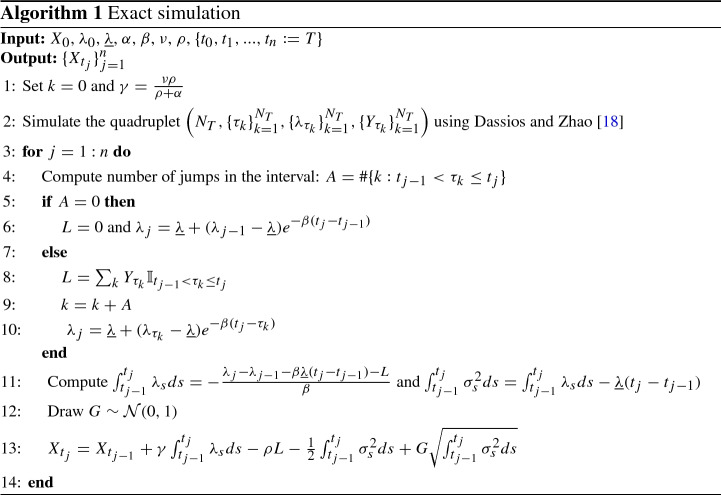


#### Remark 3

Willard [60] proposed a conditional Monte Carlo method in order to improve the efficiency of the simulation estimators under stochastic volatility models. His method is applicable to path-independent derivatives that have closed-form solutions under the Black–Scholes model and can be easily adapted to the proposed model. The first advantage of the conditional Monte Carlo approach is that European option price estimators will present a lower standard deviation, moreover, it can be used to generate unbiased estimators for the Greeks. We refer to Broadie and Kaya [12] for more details on conditional Monte Carlo formulae.

Next, we evaluate the performances of Algorithm 1 in terms of accuracy and CPU by a comparison with the Euler scheme. To do that, we follow strictly Broadie and Kaya [12]: we compute root mean square errors, $\text {RMSE} = \sqrt{\text {bias}^2 + \text {standard error}^2}$, where the bias is given by the difference between the true price of the ATM European call option computed using (12) and the simulated one. To estimate the bias precisely and remove the variance, we employ $4\times 10^8$ simulation trials for both the Euler and the exact scheme. For the Euler scheme, we consider different numbers of time discretization steps ${\mathcal {N}}$. Then, using the estimated biases, we compute RMSEs for different number of simulation trials ${\mathcal {M}}$ and record the CPU time necessary to complete the simulation. Results are displayed in Table 2 for the three different parameter sets reported in Table 1. For the exact scheme we don’t report any bias estimate since it is unbiased.

**Table 2:** Simulation results under the $\Gamma$-OU Hawkes volatility model for a European call option. Parameters are as in Table 1, top panel refers to set A (true option price: 5.6645), central panel refers to set B (true option price: 12.4740), bottom panel refers to set C (true option price: 11.7605). Other parameters: $S_0 = 100$, $K = 100$ and $T=1$. ${\mathcal {M}}\times 10^4$ is the total number of simulations, ${\mathcal {N}}\times 10^2$ is the number of time steps in the Euler scheme. Further notes: CPU is in seconds

	Euler				Exact	
${\mathcal {M}}\times 10^4$	${\mathcal {N}}\times 10^2$	bias	RMSE	CPU	RMSE	CPU
*Set A*						
1	1	0.0669	0.1000	0.16	0.0758	0.17
4	2	0.0333	0.0498	0.80	0.0375	0.38
16	4	0.0170	0.0252	6.18	0.0186	1.22
64	8	0.0092	0.0177	47.19	0.0093	4.71
256	16	0.0049	0.0068	425.33	0.0047	19.67
*Set B*						
1	1	0.1988	0.2685	0.16	0.1800	0.22
4	2	0.0996	0.1341	0.80	0.0912	0.59
16	4	0.0473	0.0652	6.14	0.0454	2.24
64	8	0.0240	0.0329	46.05	0.0226	9.16
256	16	0.0136	0.0177	421.49	0.0113	35.59
*Set C*						
1	1	0.1547	0.2672	0.16	0.2206	0.25
4	2	0.0796	0.1379	0.80	0.1124	0.72
16	4	0.0328	0.0641	6.10	0.0562	2.61
64	8	0.0179	0.0332	46.81	0.0278	10.30
256	16	0.0115	0.0181	420.54	0.0139	40.51

**Fig. 7:**
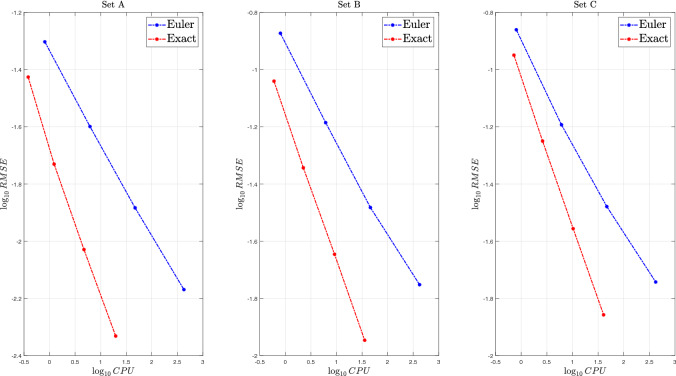
Speed-accuracy comparisons of Algorithm 1 and the Euler scheme for different parameter sets. Further notes: CPU is in seconds

Numerical results show that the exact scheme outperforms the Euler scheme both in terms of accuracy and CPU time. It presents constantly smaller RMSEs and is faster. This is particularly evident for high number of simulation trials, indeed, in the case with $256\times 10^4$ simulations, it is more than 20 times faster for parameters set A and more than 10 times faster for parameters set C than the Euler scheme. Finally, Fig. 7 presents a graphical speed accuracy comparison on a log-log scale. From this plot is possible to see that the proposed method exhibits the optimal convergence rate of 0.5 typical of the exact methods (see e.g. [12]), contrarily to the Euler scheme, whose convergence rate is smaller (around 0.32 for all the parameter sets).

## Conclusion

In this concluding section we want to provide a systematic comparison of the properties of the proposed $\Gamma$-OU Hawkes volatility model and the two most similar stochastic volatility models available in the literature, i.e. the BNS and Heston models. This comparison can be resumed in the following points.

- *Ergodic distribution of variance process* the three models share the same ergodic distribution, that is of Gamma type. However, a main difference between Heston and $\Gamma$-OU Hawkes models is that the volatility process can not reach 0. This property can be easily deduced by the evolution given by (1) with the assumption that $\lambda _0> {\underline{\lambda }}$.
- *Parsimony* Heston model is characterized by four parameters, namely long run mean, speed of mean reversion, correlation and volatility of volatility. $\Gamma$-OU BNS and Hawkes volatility models are characterised by five parameters: background intensity (playing also the role of a reverting level in the Hawkes model framework), mean reverting speed, leverage coefficient and two parameters for the jump size specifying, respectively, the exponential law intensity and a scaling factor. The scaling factor plays a minor role. Thus, we can could consider that the three models are all parsimonious.
- *Fourier–Laplace transform* the three models are exponential affine and the associated pseudo-Riccati differential equation can be solved explicitly. In particular, the inverse of the joint Fourier–Laplace transform of $(X, \lambda )$ is an explicit function containing only functions of $\ln$ and $\arctan$ type as in Heston model. It exists a huge amount of literature devoted to numerical methods for option pricing based on Fourier transform inversion methods (e.g. [15, 26]). All these methods can be adapted to the present framework of the $\Gamma$-OU Hawkes model, see Sect. 4. Moreover, it is not surprising that the term-structure and the evolution in time of the implied volatility for intermediate and longer maturities is similar to the one described by the Heston model. However, for very short maturities, the implied volatility behaviour is definitively more similar to that exhibited by the BNS $\Gamma$-OU.
- *Exact simulation scheme* we show that the proposed model can be simulated exactly without resorting to any numerical method. This is a significant advantage with respect to the Heston model whose transition can be still simulated exactly but only resorting to time consuming numerical techniques (i.e. repeated numerical inversion of Laplace transforms and root finding algorithms, see [12]). The simulation scheme we propose is unbiased and can be easily exploited not only for European options but also for more complex options such as barrier and other path dependent options. Vanilla option pricing can be performed in a very fast way also by simulating the Hawkes process and then applying a conditional Black–Scholes formula in the same spirit of Hull and White [38], Romano and Touzi [56], Willard [60] and Broadie and Kaya [12]. The same approach can be used also to obtain unbiased estimates for the Greeks.
- *Explosion of moments* $\Gamma$-OU Hawkes volatility model has not the undesirable property that moments of order higher than 1 can explode in finite time [3]. This feature is important because moment explosion can create problems in some occasions, for example when performing derivatives pricing through Monte Carlo simulation (finite second moment is necessary), or in dynamic portfolio optimization problems with power utility (the value function could be infinite when the expected value of a power of the underlying is infinite). As long as the moment generating function of the jumps size distribution exists, the moments of any order exist for all finite maturities. This is the main difference with the Heston and many other stochastic volatility models (see for instance the inverse-Gamma OU in [53]). From this point of view, $\Gamma$-OU Hawkes volatility model is more similar to $\Gamma$-OU BNS.
- *Leverage effect* it is guaranteed by the negative sign of the parameter $\rho$ multiplying the jump driver as in the usual $\Gamma$-OU BNS. In Sect. 4 we show that the slope of ATM implied volatility has a power decay as a function of the time to maturity.
- *Volatility and jumps clusters* this is the main difference between our model and the two other models. As a matter of fact, a Hawkes driver implies clusters by construction. It means that the equity prices can experience turmoil periods with spikes and high volatility levels followed by very long periods with a persistence of flat volatility.
- *Options written on VIX* $\Gamma$-OU Hawkes volatility exhibits an increasing implied volatility for options written on VIX, in coherence with empirical evidence, whereas $\Gamma$-OU BNS and Heston model are down-sloping. This result is particularly important since, comparing BNS and Hawkes volatility models, the only difference is in the self-exciting property of the jumps in Hawkes framework. An increasing implied volatility could be obtained in a Lévy framework as for instance inverse-Gamma OU in Nicolato et al. [53], but without preserving the existence of all moments.

In conclusion, the $\Gamma$-OU Hawkes volatility model introduced in this paper is parsimonious but can reproduce a broad range of empirical evidences and, in particular, the behavior of VIX options. For numerical purposes, the explicit Laplace transform enables to exploit characteristic function inversion techniques for vanilla pricing and calibration as well. An efficient exact simulation scheme can be exploited for more complex payoffs.

## Data Availability

The data used in the present paper were downloaded by using a licence issued by Bloomberg under the restriction they cannot be made public.

## References

[1] Abergel, F., Jedidi, A.: Long-time behavior of a Hawkes process-based limit order book. SIAM J. Financial Math. **6**, 1026–1043 (2015)

[2] Abi Jaber, E., Larsson, M., Pulido, S.: Affine volterra processes. Ann. Appl. Prob. **5**, 3155–3200 (2019)

[3] Andersen, L., Piterbarg, V.: Moment explosions in stochastic volatility models. Finance Stochast. **11**, 29–50 (2007)

[4] Avellaneda, M., Papanicolaou, A.: Statistics of vix futures and applications to trading volatility exchange-traded products. J. Invest. Strat. **7**, 1–33 (2018)

[5] Barndorff-Nielsen, O., Shephard, N.: Modelling by Lévy Processes for Financial EconometricsIn Lévy Processes. Birkhaüser, Boston (2001a)

[6] Barndorff-Nielsen, O., Shephard, N.: Non-Gaussian Ornstein–Uhlenbeck-based models and some of their uses in financial economics. J. R. Stat. Soc. B **63**, 167–241 (2001b)

[7] Bates, D.: Jumps and stochastic volatility: the exchange rate processes implicit in Deutsche Mark options. Rev. Financial Stud. **9**, 69–107 (1996)

[8] Bayer, C., Friz, P., Gatheral, J.: Pricing under rough volatility. Quant. Finance **16**, 887–904 (2016)

[9] Ben Dor, A., Guan, J.: Hedging systematic risk in high yield portfolios with a synthetic overlay: a comparative analysis of equity instruments vs. credit default swaps. J. Fixed Income **26**, 5–24 (2017)

[10] Bernis, G., Salhi, K., Scotti, S.: Sensitivity analysis for marked Hawkes processes: application to CLO pricing. Math. Financ. Econ. **12**, 541–559 (2018)

[11] Brignone, R., Sgarra, C.: Asian options pricing in Hawkes-type jump-diffusion models. Ann. Finance **16**, 101–119 (2020)

[12] Broadie, M., Kaya, O.: Exact simulation of stochastic volatility and other affine jump diffusion processes. Oper. Res. **54**, 217–231 (2006)

[13] Cai, N., Song, T., Chen, N.: Exact simulation of the SABR model. Oper. Res. **65**, 931–951 (2017)

[14] Callegaro, G., Gaïgi, M., Scotti, S., Sgarra, C.: Optimal investment in markets with over and under-reaction to information. Math. Financ. Econ. **11**, 299–322 (2017)

[15] Carr, P., Madan, D.: Option valuation using the fast Fourier transform. J. Comput. Finance **2**, 61–73 (1999)

[16] Chicago Board Options Exchange (2009) Cboe Volatility Index

[17] Cox, J., Ingersoll, J., Ross, S.: A theory of the term structure of interest rates. Econometrica **53**, 385–407 (1985)

[18] Dassios, A., Zhao, H.: Exact simulation of Hawkes process with exponentially decaying intensity. Electron. Commun. Prob. **18**, 1–13 (2013)

[19] Dawson, D.A., Li, Z.: Skew convolution semigroups and affine Markov processes. Ann. Probab. **34**, 1103–1142 (2006)

[20] Dawson, D.A., Li, Z.: Stochastic equations, flows and measure-valued processes. Ann. Probab. **40**, 813–857 (2012)

[21] Demeterfi, K., Derman, E., Kamal, M., Zou, J.: More than you ever wanted to know about volatility swaps. Goldman Sachs Quantitative Strategies Research Notes, March 1999, pp. 1–56 (1999)

[22] Duffie, D., Pan, J., Singleton, K.: Transform analysis and asset pricing for affine jump diffusions. Econometrica **68**, 1343–1376 (2000)

[23] El Euch, O., Fukasawa, M., Rosenbaum, M.: The microstructural foundations of leverage effect and rough volatility. Finance Stochast. **22**, 241–280 (2018)

[24] El Euch, O., Rosenbaum, M.: The characteristic function of rough Heston models. Math. Finance **19**, 3–38 (2019)

[25] Errais, E., Giesecke, K., Goldberg, L.: Affine point processes and portfolio credit risk. SIAM J. Financial Math. **1**, 642–665 (2010)

[26] Fang, F., Oosterlee, C.: A novel pricing method for European options based on Fourier-cosine series expansions. SIAM J. Sci. Comput. **31**, 826–848 (2008)

[27] Feunou, B., Okou, C.: Risk-neutral moment-based estimation of affine option pricing models. J. Appl. Economet. **33**, 1007–1025 (2018)

[28] Fičura, M.: Forecasting jumps in the intraday foreign exchange rate time series with hawkes processes and logistic regression. In: Procházka, D. (ed.) New Trends in Finance and Accounting. Springer, New York (2017)

[29] Gonzato, L.: Application of Sequential Monte Carlo Methods to Dynamic Asset Pricing Models. Dissertation, University of Milano-Bicocca (2020)

[30] Granelli, A., Veraart, A.: Modeling the variance risk premium of equity indices: the role of dependence and contagion. SIAM J. Financial Math. **7**, 382–417 (2016)

[31] Hagan, P.S., Kumar, D., Lesniewski, A.S., Woodward, D.E.: Managing smile risk. Wilmott Magazine, pp. 84–108, (2002)

[32] Hainaut, D.: A model for interest rates with clustering effects. Quant. Finance **16**, 1203–1218 (2016)

[33] Hawkes, A.G.: Spectra of some self-exciting and mutually exciting point processes. Biometrika **58**, 83–90 (1971)

[34] Heston, S.: A closed-form solution for options with stochastic volatility with applications to bond and currency options. Rev. Financial Stud. **6**, 327–343 (1993)

[35] Horst, U., Xu, W.: The microstructure of stochastic volatility models with self-exciting jump dynamics. Preprint arXiv: https://arxiv.org/abs/1911.12969 (2019a)

[36] Horst, U., Xu, W.: A scaling limit for limit order books driven by Hawkes processes. SIAM J. Financial Math. **10**, 350–393 (2019b)

[37] Hubalek, F., Sgarra, C.: On the Esscher transforms and other equivalent martingale measures for Barndorff–Nielsen and Shephard stochastic volatility models with jumps. Stoch. Process. Their Appl. **119**, 2137–2157 (2009)

[38] Hull, J., White, A.: Pricing interest-rate derivative securities. Rev. Financial Stud. **3**, 573–592 (1990)

[39] Jiao, Y., Ma, C., Scotti, S.: Alpha-CIR model with branching processes in sovereign interest rate modeling. Finance Stochast. **21**, 789–813 (2017)

[40] Jiao, Y., Ma, C., Scotti, S., Sgarra, C.: A branching process approach to power markets. Energy Economics **79**, 144–156 (2019a)

[41] Jiao, Y., Ma, C., Scotti, S., Zhou, C.: The alpha–Heston volatility model. Forthcoming on Math. Finance. Preprint Arxiv: https://arxiv.org/abs/1812.01914 (2019b)

[42] Kallsen, J., Muhle-Karbe, J.: Exponentially affine martingales, affine measure changes and exponential moments of affine processes. Stoch. Process. Appl. **120**, 163–181 (2010)

[43] Kallsen, J., Muhle-Karbe, J., Vo, M.: Pricing option on variance in affine stochastic volatility models. Math. Finance **21**, 627–641 (2011)

[44] Kallsen, J., Shiryaev, A.: The cumulant process and the Esscher change of measure. Finance Stoch. **6**, 397–428 (2002)

[45] Keller-Ressel, M.: Moments explosions and long-term behavior of affine stochastic volatility models. Math. Finance **21**, 73–98 (2011)

[46] Kienitz, J., Wetterau, D.: Financial Modelling—Theory, Implementation and Practice with Matlab, 2 edn. Wiley Finance Series (2012)

[47] Kou, S.: A jump-diffusion model for option pricing. Manag. Sci. **48**, 1086–1101 (2002)

[48] Kou, S.: Jump-diffusion models for asset pricing in financial engineering. In: Birge, J., Linetsky, V. (eds.) Handbooks in OR & MS, vol. 15, pp. 73–116. Elsevier (2008)

[49] Li, C., Wu, L.: Exact simulation of the Ornstein–Uhlenbeck driven stochastic volatility model. Eur. J. Oper. Res. **275**, 768–779 (2019)

[50] Li, Z.: Measure-Valued Branching Markov Processes. Probability and Its Applications. Springer, Belin (2011)

[51] Li, Z., Ma, C.: Catalytic discrete state branching models and related limit theorems. J. Theor. Probab. **21**, 936–965 (2008)

[52] Muhle-Karbe, J., Pfaffel, O., Stelzer, R.: Option pricing in multivariate stochastic volatility models of OU type. SIAM J. Financial Math. **3**, 66–94 (2011)

[53] Nicolato, E., Pisani, C., Sloth, D.: The impact of jump distributions on the implied volatility of variance. SIAM J. Financial Math. **8**, 28–53 (2017)

[54] Nicolato, E., Venardos, E.: Option pricing in stochastic volatility models of the Ornstein–Uhlenbeck type. Math. Finance **13**, 445–466 (2003)

[55] Rhoads, R.: Trading VIX Derivatives: Trading and Hedging Strategies Using VIX Futures, Options, and Exchange-Traded Notes. Wiley Trading Series. Wiley, Hoboken (2011)

[56] Romano, M., Touzi, N.: Contingent claims and market completeness in a stochastic volatility model. Math. Finance **7**, 399–412 (1997)

[57] Schöbel, R., Zhu, J.: Stochastic volatility with an Ornstein–Uhlenbeck process: An extension. Eur. Finance Rev. **3**, 23–46 (1999)

[58] Sepp, A.: Pricing options on realized variance in the Heston model with jumps in returns and volatility. J. Comput. Finance **11**, 33–70 (2008)

[59] Veraart, A., Veraart, L.: Stochastic volatility and stochastic leverage. Ann. Finance **8**, 205–233 (2012)

[60] Willard, G.A.: Calculating prices and sensitivities for path-independent derivative securities in multifactor models. J. Derivatives **5**, 54–61 (1997)

[61] Zheng, B., Roueff, F., Abergel, F.: Modelling bid and ask prices using constrained Hawkes processes: ergodicity and scaling limit. SIAM J. Financial Math. **5**, 99–136 (2014)

